# Implementation of the ‘Countdown to Theatre’ Approach to Bridge the Evidence–Practice Gap in Paediatric Preoperative Fasting: A Quality Improvement Initiative

**DOI:** 10.1111/jan.70162

**Published:** 2025-08-27

**Authors:** Erika Dulay, Bronwyn Griffin, Du Trung, Craig A. Mcbride, Adrienne P. Hudson, Diana Hermith‐Ramirez, Amanda J. Ullman

**Affiliations:** ^1^ Children's Health Queensland Hospital and Health Service South Brisbane Queensland Australia; ^2^ School of Nursing and Midwifery Griffith University Brisbane Queensland Australia; ^3^ Children's Health Research Centre The University of Queensland Brisbane Queensland Australia; ^4^ School of Nursing, Midwifery and Social Work The University of Queensland Brisbane Queensland Australia; ^5^ Griffith Biostatistics Unit, Griffith Health Griffith University Brisbane Queensland Australia

**Keywords:** children, fasting, implementation science, preoperative

## Abstract

**Aim:**

To evaluate the ‘Countdown to Theatre’ intervention, a co‐designed nurse‐led approach developed using the COM‐B framework to address context‐specific barriers and facilitators to preoperative fasting practices.

**Design:**

A prospective mixed‐method, pre–post study assessed the intervention's impact on fasting adherence and patient experience.

**Methods:**

Participants included children booked for a procedure under general anaesthesia. Adherence was assessed through audited fasting duration, and patient experience was evaluated using caregiver/patient surveys. The intervention was implemented and monitored by nursing staff as a part of a structured quality improvement process. Nurses played a central role in embedding the approach into daily workflows and reinforcing fasting timelines

**Results:**

Over 9 months, 901 observations were undertaken from 774 patients. Fasting duration decreased from 7.6 to 5.7 h (mean difference −1.94; 95% CI −3.04, −0.86). Parent‐reported patient experience surveys showed improvement in many areas, including an increase in overall satisfaction (from 44.7% to 68.8%).

**Conclusion:**

The intervention successfully reduced prolonged fasting and improved patient experiences, demonstrating the value of co‐designed approaches in addressing evidence–practice gaps in perioperative care.

**Implications for Patient Care:**

The principles of co‐design, structured implementation and the application of the COM‐B framework provide a replicable model for addressing similar challenges in healthcare. The study highlights the pivotal role of nurses in improving perioperative practices, supporting both patient safety and satisfaction. Future research should explore the intervention's applicability across diverse settings and patient populations.

**Impact:**

Despite evidence‐based guidelines, excessive preoperative fasting remains prevalent in practice. This study demonstrates that a structured, nurse‐led intervention can successfully reduce fasting durations and enhance patient experience, reaffirming the nursing profession's capacity to lead meaningful change in perioperative care.

**Reporting Method:**

Standards for quality improvement reporting excellence (SQUIRE 2.0).

**Patient or Public Contribution:**

Patients and caregivers contributed to the co‐design of the intervention, ensuring that it addressed practical challenges related to preoperative fasting.

**Summary**

- Prolonged preoperative fasting remains a persistent challenge in surgical care, reflecting an ongoing gap between evidence and practice.
- This study demonstrates that a structured, nurse‐led intervention can successfully reduce fasting durations and enhance patient experience, reaffirming the nursing profession's capacity to lead meaningful change in perioperative care.
- While this study implemented a context‐specific intervention, its co‐design approach and application of the COM‐B framework provide a practical, replicable model for healthcare settings seeking to address similar practice gaps through structured, behaviour‐informed implementation.

## 1 Introduction

Prolonged preoperative fasting remains a significant challenge in surgical care, with pronounced implications in paediatric populations (Dulay et al. 2024). Originally designed as a precaution against pulmonary aspiration during anaesthesia (Frykholm et al. 2018; He et al. 2022; Mesbah and Thomas 2017), historical fasting protocols, such as the traditional ‘nil by mouth after midnight' policy, were founded on highly conservative 'safety first' principles (Chon et al. 2017; Rüggeberg et al. 2024). While these protocols maximised airway safety and ensured operational efficiency by maintaining patient readiness for theatre with fasted patients, they often resulted in fasting periods long exceeding what was clinically necessary. As growing evidence underscored the adverse effects of prolonged fasting, research into the kinetics of gastric emptying gained prominence (Frykholm et al. 2018; Mesbah and Thomas 2017; Rüggeberg et al. 2024). This body of work has supported the safe reduction in the required fasting duration (Andersson et al. 2018; Carvalho et al. 2017). Current guidelines now recommend fasting durations based on the type of ingested substance, typically advocating fasting times of 6‐h for food, 4‐h for breastmilk and 1‐h for clear fluids (6–4–1) (Zhang et al. 2023). The challenge remains in successfully implementing fasting protocols which minimise the risk of pulmonary aspiration, while sparing children from prolonged fasting.

### 1.1 Problem Description

Despite increased leniency in preoperative fasting guideline recommendations, a lack of translation into practice is evident (Dulay et al. 2024; Frykholm et al. 2018). Clinical audits in paediatric settings reveal most patients are fasting for excessively long periods, often up to twice the recommended length (Andersson et al. 2018; Aroonpruksakul et al. 2023; Frykholm et al. 2018; Ricci et al. 2024). This affects the physical and psychological well‐being of these children and adversely impacts the caregivers responsible for their care (Frykholm et al. 2018; Zhang et al. 2023).

Children are disproportionately affected by prolonged fasting periods. Physically immature organ systems and limited energy reserves reduce their capacity for prolonged fasting (Carvalho et al. 2017; Frykholm et al. 2018; Li et al. 2020). A faster metabolic rate and increased nutritional demands required for healthy growth and development put children at higher risk for hypoglycaemia, dehydration and other associated metabolic sequelae (Li et al. 2020). Younger and developmentally delayed children are less likely to comprehend the necessity of fasting, thus leading to irritability, crying, and an increased likelihood of caregiver noncompliance (Frykholm et al. 2018; Li et al. 2020). This is a particular problem for breastfed children who can become even more distressed with the caregiver's attempts to settle them, as they can sense the presence of what they desire but are not permitted to have.

The stress and anxiety associated with managing a child's fasting regimen can strain familial relationships and increase parental distress (Frykholm et al. 2018; Zhang et al. 2023). These negative preoperative experiences highlight the need to bridge the gap between evidence‐based recommendations and clinical practice.

### 1.2 Available Knowledge

Globally, interventions to reduce prolonged fasting have included changes in fasting protocols, processes to improve communication, uses of technology, individualised fasting programmes and staff education (Andersson et al. 2018; Buller and Sims 2016; Carroll et al. 2022; Dennhardt et al. 2016; Downie et al. 2019; Dulay et al. 2024; Isserman et al. 2019; Li et al. 2020; Newton et al. 2017; Nye et al. 2019; Ong and Walker 2022; Rawlani et al. 2022; Thomasseau et al. 2021). However, the success of these interventions often depends on their ability to address local barriers and facilitators (Dulay et al. 2024). Interventions working to instil confidence in clinicians or family members to regularly offer fluids, while adhering to fasting recommendations, showed a reduction in actual fasting duration without compromising patient safety (Dulay et al. 2024). Poor intervention design with poor stakeholder engagement often led to intervention and implementation failure (Dulay et al. 2024).

### 1.3 Rationale

To address the problem of prolonged preoperative fasting in paediatric clinical practice, this study employed the COM‐B Behaviour Change Wheel framework, which posits that successful behaviour change hinges on three essential conditions (Michie et al. 2011). The patient, caregiver or clinician must have:

1. The physical and psychological Capability (C) to carry out the behaviour,
2. The social and physical Opportunity (O) to engage in the behaviour and
3. The Motivation (M) to perform the Behaviour (B).

Using this implementation science approach, the study identified context‐specific barriers and facilitators impacting adherence to fasting guidelines and contributing to prolonged preoperative fasting. The implementation strategy was co‐designed to address these barriers and leverage the facilitators, thereby enhancing the likelihood of implementation success.

### 1.4 Specific Aims

This study aimed to (a) uncover barriers and facilitators to fasting guideline adherence, to inform the (b) implementation of co‐designed interventions and ultimately (c) improve fasting guideline adherence and patient and family experiences preoperatively. The research questions were as follows:

1. What practice barriers perpetuate prolonged preoperative fasting, and what facilitators support adherence to optimal fasting, as identified by parents and clinicians?
2. How did fasting guideline adherence and use of intravenous fluid therapy change immediately, at 3 months and at 6 months following intervention implementation?
3. How does patient experience change after the intervention was implemented?

## 2 Methods

### 2.1 Context

This single‐centre study was conducted within the Surgical Division at Queensland Children's Hospital (QCH) between June 2022 and March 2023. QCH, which provides specialist healthcare services to children from birth to 18 years of age (Government Q. Queensland Children's Hospital 2024), has had a 6–4–1 fasting policy in place since 2019.

This study included acute and emergency patients undergoing a procedure under general anaesthesia, specifically those scheduled on the hospital's acute surgical list, commonly referred to as the ‘Emergency Board’. This list is reserved for nonelective patients requiring urgent or semiurgent procedures and typically comprises children admitted through the Emergency Department or referred for unplanned surgical care. The Emergency Board typically ran from 8:00 AM until 11:00 PM, adopting a ‘life or limb’ assessment strategy regarding overnight operating. These procedures are conducted based on clinical priority rather than scheduled time slots. As a result, patients on this list are not given specific theatre times, and their expected time to theatre often fluctuates depending on the volume, complexity and clinical urgency of other cases on the surgical list that day. This population was intentionally chosen as they are particularly vulnerable to prolonged fasting, given the unpredictable and prioritised nature of theatre scheduling on the Emergency Board.

Elective patients were excluded as this cohort is typically given specific fasting instructions based on their booked theatre time. Patients who do not normally consume food or drinks orally, who were fasting due to clinical need rather than for a procedure under general anaesthesia, or who had a pre‐existing medical condition that increased the potential to delay gastric emptying were also excluded.

### 2.2 Study Design

A prospective mixed‐method, pre–post study was conducted over three distinct phases.

#### 2.2.1 Phase I—Preintervention Baseline (June–July 2022)

In Phase 1, preintervention adherence and caregiver assessment of patient experience were collected:

- Adherence: The primary outcome measure for adherence was fasting duration collected through an audit of the electronic medical records (Cerner ieMR). Secondary measures included the occurrence of intravenous (IV) fluid therapy administration and the need for a fluid bolus either pre‐ or postoperatively.
- Patient experience: A purpose‐built electronic survey was used to assess the overall preoperative fasting experience. This survey was disseminated by bedside nursing staff using a QR code linking to a Microsoft Forms Survey and was completed by parents or caregivers on behalf of paediatric patients.

#### 2.2.2 Phase II—Stakeholder Co‐Design and Intervention Implementation (August 2022)

In Phase 2, barriers and facilitators to prolonged fasting were identified:

- Survey: Within the patient experience survey, parents were asked about their experiences of fasting practices to help inform intervention design.
- Clinician focus groups: Focus groups were conducted with purposively selected clinicians representing anaesthesia, surgery, nursing, dietetics, patient safety, clinical education and nursing leadership. Participants were identified through consultation with department heads to ensure representation across a broad range of roles in perioperative care.
  Focus groups were semistructured and guided by the COM‐B framework, using a set of open‐ended prompts to direct discussion while allowing the flexibility to explore emerging themes. Sessions were facilitated by a member of the research team, with oversight from an experienced qualitative researcher.
- Co‐design: Following the identification of key barriers and facilitators, the same multidisciplinary stakeholders engaged in a collaborative co‐design process to develop tailored intervention strategies. This involved joint brainstorming and iterative refinement to ensure that the proposed intervention prioritised patient safety, demonstrated feasibility with existing clinical workflows, and mitigated the potential for surgical delays.
- Implementation and Roll‐out: The intervention was implemented through a comprehensive, multimodal education strategy primarily targeting bedside nursing staff and theatre coordinators, who were pivotal to its execution. This included structured, in‐person in‐service training sessions that conveyed the rationale, objectives, and procedural changes associated with the intervention. Nurse Unit Managers (NUMs) played a key role in facilitating dissemination by circulating detailed intervention materials via email to their respective teams, thereby ensuring broad awareness and engagement.
  Supplementary printed materials summarising the intervention were strategically placed in nursing break rooms, and informational posters were displayed on departmental education boards to provide ongoing visual reinforcement. During the two‐week implementation phase, the research team conducted frequent rounds across clinical areas to offer real‐time support, respond to staff inquiries, deliver supplemental education to personnel who had not attended formal sessions, and address any emergent concerns. This proactive engagement was critical to fostering staff confidence, promoting adherence to the new protocol and facilitating integration within existing clinical workflows.

#### 2.2.3 Phase III—Postintervention Evaluation (September 2022—March 2023)

In Phase 3, the interventions were implemented on Monday, 5 September 2022, and their impact on adherence was evaluated over 3 time periods: immediately (Post Phase I: 5–30 September 2002), 3 months after implementation (Post Phase II: 1–31 December 2022) and 6 months after implementation (Post Phase III: 1–31 March 2023). These intervals were selected to assess both the immediate effects and the longer‐term sustainability of the intervention. Patient experience surveys were collected preintervention and immediately postintervention (Post‐Phase I). Due to funding limitations, follow‐up surveys at subsequent time points were not conducted, thereby concentrating the assessment on baseline and immediate postintervention patient‐reported outcomes.

### 2.3 Interventions

The core intervention comprised a structured and safe reallocation of responsibility for offering clear fluids, shifting this role from the theatre coordinators—who often had limited visibility of individual patient fasting status and its associated sequelae—to bedside nursing staff. This change empowered nurses providing direct preoperative care to take a more proactive and central role in managing patients' fasting experiences, while also supporting theatre coordinators in effectively managing the surgical schedule through improved transparency of fasting status. To support this shift, nurses were equipped with clear co‐designed guidance, referred to as ‘Countdown to Theatre’.

This intervention was developed to align with existing fasting policies while proactively addressing local barriers and facilitators contributing to prolonged fasting. Its goal was to strike an effective balance between ensuring patient safety through appropriate fasting and alleviating the physiological and emotional burden of prolonged fasting. In doing so, the intervention aimed to improve overall patient comfort and experience, without contributing to surgical delays.

**FIGURE 1:**
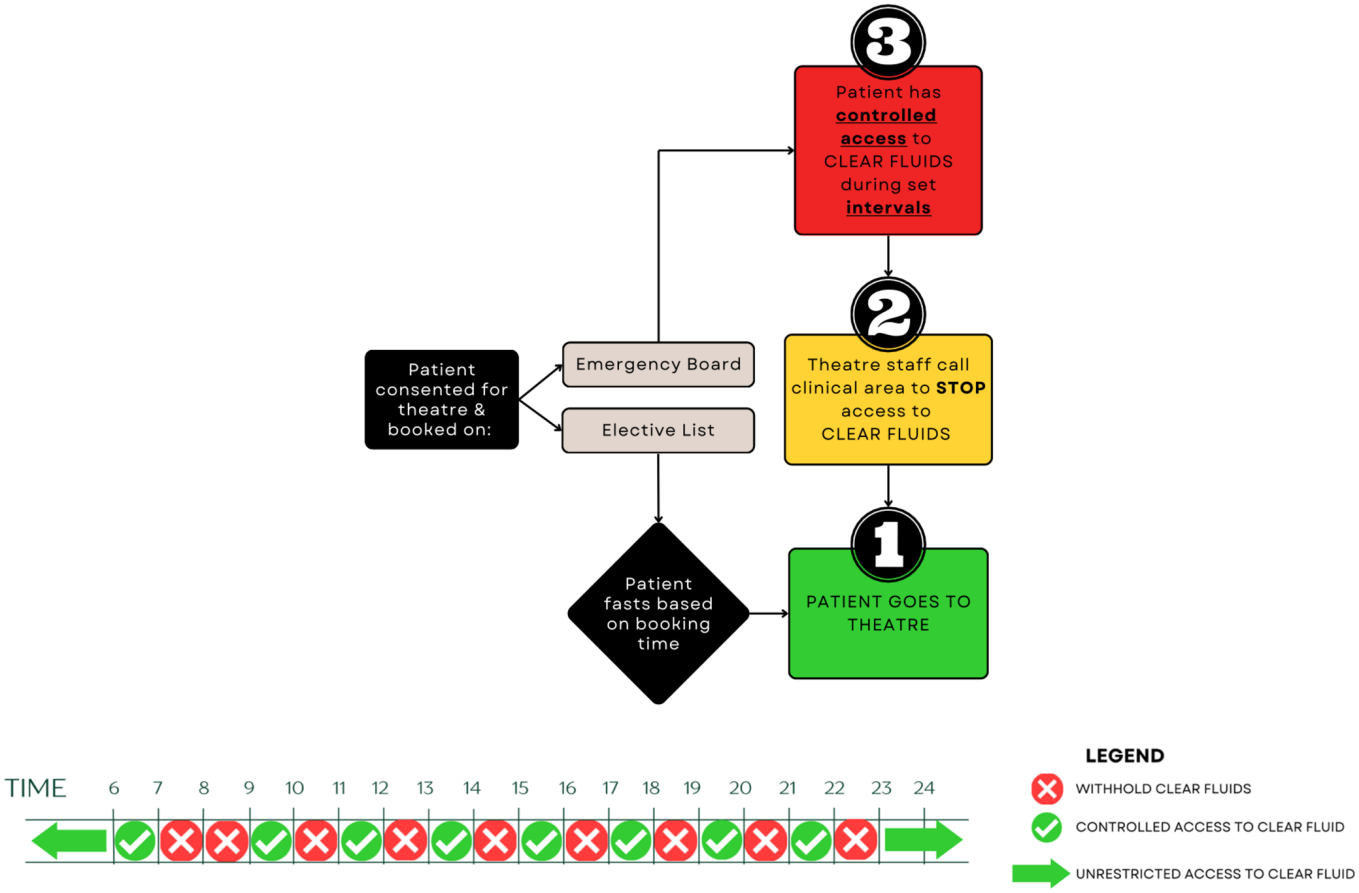
Countdown to theatre intervention.

To translate the intervention into practice, a co‐design process was employed, following the findings of Phase II, to develop targeted interventions aimed at addressing the identified barriers and facilitators. These included educational initiatives (Figure S1) and support resources (Figure S2). All patients scheduled on the Emergency Board followed the ‘Countdown to Theatre’ intervention displayed in Figure 1. This three‐stage intervention was designed to ensure that patients were appropriately fasted and ready for surgery, concurrently prioritising patient safety, minimising unnecessary delays and optimising patient comfort.

All Emergency Board patients commenced their fast at Stage 3 (Figure 1). During this stage, patients had controlled access to clear fluids during set intervals. Bedside nurses were responsible for controlling:

-
*Type of Fluid:* Only ‘Clear Fluid’ was allowed, including water, pulp‐free clear apple juice, clear diluted cordial, electrolyte drinks, or reconstituted ready‐to‐mix electrolyte powders. Carbonated fluids or those containing milk, food fibre (e.g., pulp) or thickeners were excluded.
-
*Amount of Fluid:* Patients could consume up to 3 mL/kg per serve. As a general guide, patients between 1 and 5 years of age were allowed up to 50 mL; 6–10 years were allowed up to 100 mL; and > 10 years were allowed up to 150 mL.
- *Timing:* At the beginning of each ‘allowed to drink’ hour (indicated by a ‘green tick’ in Figure 1) bedside nurses offered patients a serve of clear fluids to consume within the designated hour. Any fluids not consumed within the designated hour were removed to ensure adherence to the fasting schedule.
  To align with the start of surgical operations at 8:00 AM, a 2‐hour ‘no drink period’ (as indicated by the red crosses in Figure 1) was employed to ensure that all patients were fasted when theatres commenced, thereby promoting smooth patient flow at the start of the day. The ‘no drink period’ occurred again every second hour after 9:00 AM to give theatre coordinators clear visibility of patients' fasting status at all times, as all patients would be adequately fasted at the end of each ‘no drink’ hour.
  From 11:00 PM until 06:00 AM the following day, all patients had unrestricted access to clear fluids unless theatre coordinators indicated specific patients were still scheduled for their procedure during that time period.

A patient transitioned to Stage 2 when the theatre coordinator notified bedside clinicians that theatre was imminent (i.e., within the next hour). At this stage, all access to clear fluids was discontinued and no further oral fluids were provided unless explicitly directed by the theatre coordinator. Bedside clinicians were instructed to inform patients and families, update the preoperative checklist documentation and ensure that the patient was ready for transfer.

If a delay occurred after moving a patient to Stage 2, it was the responsibility of the theatre coordinator to notify bedside clinicians and revert the patient back to Stage 3. Once the theatre team was ready to receive the patient, arrangements were made for their transport to theatre, transitioning them to Stage 1.

Patients on the Emergency Board cohort could remain engaged with the ‘Countdown to Theatre’ intervention for durations as little as 1h to an entire day (8 AM–11 PM). Importantly, the intervention did not alter surgical wait times but was implemented concurrently with existing surgical workflows. Consequently, there was no standardised duration for engagement with the intervention, as individual patient experiences varied considerably based on the volume, complexity and clinical urgency of the daily surgical caseload.

Once a patient was consented and assigned to the Emergency Board, they adhered to the ‘Countdown to Theatre’ protocol alongside other patients. Should a patient be placed on the board during a designated ‘no drink’ interval, fasting commenced immediately, with clear fluids permitted only at the subsequent ‘drink‐allowed’ interval, as delineated by the timing schedule in Figure 1. In situations where the surgical team deemed the procedure urgent and imminent, existing verbal communication channels were maintained, overriding the timing protocol, thereby preserving flexibility while prioritising patient safety.

### 2.4 Study of the Interventions

Intervention effectiveness was evaluated through a combination of adherence and patient experience measures. Adherence data were collected at baseline (preimplementation) and three distinct intervals postimplementation: immediately (Post‐Phase I), 3 months post (Post‐Phase II) and 6 months post (Post‐Phase III).

### 2.5 Adherence Measures

Fasting metrics for this study were documented as part of standard care. A Children's Health Queensland (CHQHHS 2023) customised ‘Fasting Report’ was generated and filtered to capture data on all eligible procedures during the relevant pre‐ and postintervention phases. This report retrieved key information regarding the type and timing of intravenous therapy administered, the documented time of last oral intake and the time anaesthesia was administered. The data were then exported to Microsoft Excel (Microsoft Excel 2018) for data cleaning and patient de‐identification.

Fasting duration was calculated by subtracting the time of the last drink consumed, as documented on the preoperative checklist, from the time the anaesthesia was administered, as documented on the patient's medication administration record. This approach reflected the actual duration of preoperative fasting experienced by the patient, rather than the total time spent waiting for surgery. This metric was intentionally chosen as the aim of the intervention was not to reduce the time spent waiting for surgery, which represented a factor largely influenced by systemic and operational factors beyond the direct control of the clinical team, but to minimise the physiological and emotional burden of prolonged fasting by improving the frequency and timing of oral intake opportunities.

In instances where essential data for calculating adherence measures were missing from the automated Fasting Report, a manual audit of the patient's electronic medical record was conducted. If the required information could not be reliably ascertained, the corresponding patient encounter was excluded from the analysis. This process safeguarded the validity and consistency of adherence calculations. A comprehensive list of excluded encounters, along with the specific reasons for exclusion, is provided in Figure 3.

### 2.6 Patient Experience Measures

Patient experience was captured using a purpose‐built, multimethod survey tool. The survey included nine questions, incorporating both open‐ and closed‐ended responses. Five‐point Likert items (1—very unsatisfied, 2—unsatisfied, 3—neutral, 4—satisfied and 5—very satisfied) were used to evaluate satisfaction with the fasting experience provided at QCH. The survey was piloted with three families who had prior experience with multiple preoperative fasting episodes to ensure clarity and relevance.

Eligible participants were identified via convenience sampling during both the preintervention phase and the immediately postintervention period (Post Phase I). Nursing staff within the Surgical Division distributed the survey in person to parents or caregivers present at the bedside of paediatric patients who were either actively fasting in preparation for surgery or had just completed their procedure. Participation was voluntary, and inclusion criteria required that the caregiver had accompanied the child through the fasting period on the day of survey distribution.

### 2.7 Analysis

Adherence data were thoroughly checked, cleaned and validated by a nurse researcher (ED). All data were analysed using Stata v18.0 and Python 3.11 by the study's biostatistician (DH‐R). The validation process included confirming that all audited patients met eligibility criteria, verifying the completeness of key adherence variables and confirming the accuracy of fasting duration calculations.

Descriptive statistics were used to summarise patient demographics, ASA classification, admission type, surgical specialty and fasting durations. Continuous variables such as age and fasting time were reported as means with standard deviations, and where appropriate, as medians and ranges. Categorical variables were summarised as counts and percentages.

For the primary outcome, fasting duration in hours, a linear mixed‐effects model was fitted to account for repeated measures from the same individuals across the four study timepoints (preintervention, post‐Phase I, post‐Phase II, post‐Phase III). *Timepoint* was included as a categorical

fixed effect, and *patient identifier* as a random intercept to account for within‐subject correlation.

Covariates such as age group, ASA classification and admission type were considered for inclusion but ultimately not retained, as there were no systematic differences in these characteristics across timepoints. As such, adjustment for confounding was deemed unnecessary. Residuals were checked for normality and homoscedasticity, and no major violations were identified. The inclusion of random effects improved model fit, supported by intraclass correlation estimates. Model assumptions were checked and confirmed.

Secondary outcomes included: Fasting adherence category, defined as ‘Good’ (1–2 h), ‘Average’ (3–5 h) and ‘Poor’ (> 6 h); IV fluid administration, evaluated across the pre‐, intra‐ and postoperative periods. Fasting adherence was analysed using multinomial logistic regression, with timepoint as the sole explanatory variable. This model estimated the odds of being in the ‘Average’ or ‘Poor’ category compared to the reference category (‘Good’).

IV fluid therapy and IV fluid bolus administration were analysed using binary logistic regression; also, timepoint was the only predictor. These models were designed to assess temporal trends following the intervention. Adjustment for confounding was not necessary, as the populations did not differ systematically across timepoints.

Goodness‐of‐fit tests (e.g., Pearson chi‐square) were performed for all models, and assumptions such as multicollinearity and separation were evaluated and met. Model assumptions were confirmed for all regression analyses.

These models were prespecified. As the study was exploratory and focused on descriptive temporal trends rather than formal hypothesis testing, no corrections for multiple comparisons were applied.

Open‐ended patient experience survey responses underwent reflexive thematic analysis to deductively identify codes and analyse themes related to fasting practice satisfaction (Braun and Clarke 2006; Braun and Clarke 2024). This involved key steps including: 1. Getting familiar with the data; 2. Generating initial codes; 3. Searching for themes; 4. Reviewing themes; 5. Defining and naming themes; and 6. Producing the report (Braun and Clarke 2006). In addition, the closed‐ended survey responses were descriptively analysed.

### 2.8 Ethical Considerations

The project was reviewed by the CHQ Human Research Ethics Committee prior to commencement and granted an exemption under the terms of quality improvement (EX/22/QCHQ/84401) (NHMRC 2024).

## 3 Results

### 3.1 Barriers and Facilitators to Preoperative Fasting Adherence

Of the 11 clinicians participating in the focus group discussions and the 47 parents completing the surveys, several barriers and facilitators were descriptively identified as influencing adherence to preoperative fasting guidelines. These were categorised in line with the COM‐B Implementation Science Framework (Michie et al. 2011) to inform intervention design summarised in Table 1.

**TABLE 1:** Barriers and facilitators to fasting adherence using the COM‐B framework.

	Barriers to adherence	Facilitators to adherence
**Capability** *‘Physical or psychological capability to engage in the behaviour’*	**Lack of knowledge**: Parents and caregivers received limited education on the preoperative fasting requirements **Lack of patient involvement**: age‐appropriate patients were rarely involved in education on fasting requirements	**Understanding of fasting requirements:** Clinicians had a clear and thorough understanding of fasting requirements **Recognition of excessive fasting symptoms**: Clinicians were well equipped to recognise symptoms associated with excessive fasting
**Opportunity** *‘Factors that lie outside the individual that make the behaviour possible, or prompt it’*	**Inconsistent communication**: The ability to offer clear fluids was only prompted by inconsistent communication between bedside and theatre clinicians **Limited Monitoring**: There was poor visibility of patient fasting duration in real‐time	**Physical availability of clear fluids**: Clear fluids were readily available on each ward where patients were fasting for theatre **Liberal Fasting Protocol**: At QCH, the clear fluid fasting requirement was 1 h prior to anaesthesia **Rapport with Nursing Staff:** Parents report strong rapport and trust with bedside clinicians
**Motivation** *‘Habitual processes, emotional responses and analytical decision‐making that directs behaviour’*	**Fear of noncompliance**: Risks (e.g., pulmonary aspiration) of noncompliance were heavily emphasised to parents and clinicians **Lack of awareness of risks**: Insufficient emphasis was placed on the risks associated with prolonged fasting **Pressure to avoid empty theatres**: Clinicians were expected to prevent delays or cancellations to theatre due to cost concerns and failure to meet theatre key performance indicator (KPI) metrics	**Patient distress prompting parental advocacy**: The emotional response of parents to their child's distress related to fasting often motivated them to advocate for the administration of oral fluids **Supportive culture and leadership**: QCH fosters a culture that prioritises patient‐centred care and actively supports quality improvement initiatives **Understanding of fasting necessity:** Caregivers clearly understood the importance of fasting adherence for patient safety

### 3.2 Sample Characteristics

As illustrated in Table 2, the demographic and clinical characteristics of the patients were assessed at four intervals: Preintervention, Post‐Phase I, Post‐Phase II and Post‐Phase III. The average age of patients slightly increased from 7.4 to 8.2 years over this period, with school‐aged children (5–12 years) consistently making up the largest category. Both gender distribution and procedure case specialty fluctuated over time. These trends illustrate the changing patient demographics and clinical characteristics throughout the intervention phases.

**TABLE 2:** Patient demographic and clinical characteristics.

Characteristic	Preintervention (*N* = 220)	Post‐Phase I (*N* = 196)	Post‐Phase II (*N* = 222)	Post‐Phase III (*N* = 263)
Age (years)				
Mean ± SD	7.4 ± 4.7	7.5 ± 4.8	7.7 ± 4.5	8.2 ± 4.7
Age group in years (*n* (%))				
Infant < 1y	7 (3.2)	3 (1.5)	1 (0.4)	0 (0)
Toddler 1–3	59 (26.8)	58 (29.6)	52 (23.4)	57 (21.7)
Preschooler 3–5	24 (10.9)	13 (6.6)	29 (13.1)	33 (12.5)
School‐aged 5–12	88 (40)	81 (41.3)	99 (44.6)	109 (41.4)
Teenager > 12	42 (19.1)	41 (20.9)	41 (18.5)	64 (24.3)
Sex (*n* (%))				
Female	87 (39.6)	79 (40.3)	74 (33.3)	105 (39.9)
Male	133 (60.4)	117 (59.7)	148 (66.7)	158 (60.1)
ASA status (*n* (%))				
I	157 (71.4)	129 (65.8)	166 (74.8)	196 (74.5)
II	37 (16.8)	48 (24.5)	37 (16.7)	48 (18.3)
III	26 (11.8)	19 (9.7)	17 (7.7)	19 (7.2)
IV	—	—	2 (0.9)	—
Admission type (*n* (%))				
Day case	1 (0.4)	6 (3.1)	3 (1.4)	5 (1.9)
Day of surgery admission	142 (64.6)	134 (68.4)	175 (78.8)	193 (73.4)
Inpatient	77 (35)	56 (28.6)	43 (19.4)	65 (24.7)
Outpatient	—	—	1 (0.4)	—
Case specialty (*n* (%))				
General Surgery	2 (0.9)	2 (1.0)	1 (0.4)	4 (1.5)
Orthopaedic	94 (42.7)	63 (32.1)	82 (36.9)	83 (31.6)
Ear Nose Throat	17 (7.7)	16 (8.2)	20 (9.0)	12 (4.6)
Plastics	26 (11.8)	35 (17.9)	22 (9.9)	45 (17.1)
Other	81 (36.8)	80 (40.8)	97 (43.7)	119 (45.2)

*Note:* Standard Deviation (±SD).

### 3.3 Preoperative Fasting Adherence

**FIGURE 2:**
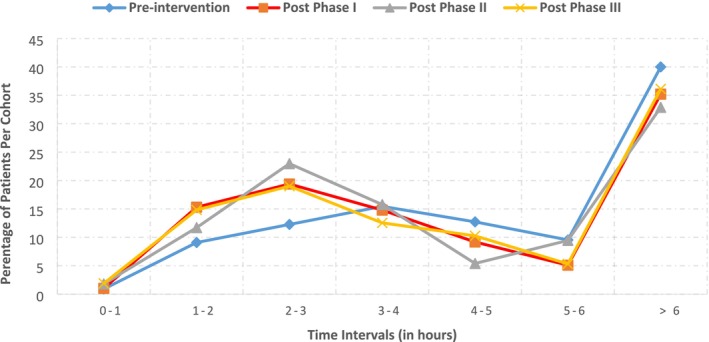
Fasting adherence by time interval.

Preoperative fasting adherence was observed across the preintervention and all postintervention phases, consisting of 901 observations from 774 unique patients. A reduction in mean fasting duration was observed at all postintervention time points compared to the preintervention period, as shown in Table 3 and Figure 2. The most pronounced changes were evident at 6 months postintervention (Post Phase III). Good adherence to guidelines, measured by a clear fluid fasting duration of 1–2 h preanaesthesia, rose from 9.1% preintervention to 15.3% Post‐Phase I and remained relatively high at 14.8% in Post‐Phase III. Average adherence, defined as a fasting duration between 3 and 5 h, remained consistent across all phases, while poor adherence, measured by a fasting duration greater than 6 h, declined in all postimplementation phases.

**TABLE 3:** Adherence measures to preoperative fasting guidelines.

Measures	Preintervention	Post‐Phase I	Post‐Phase II	Post‐Phase III	Preintervention vs. Post‐Phase I	Preintervention vs. Post‐Phase II	Preintervention vs. Post‐Phase III
					Mean difference (95% CI)	Mean difference (95% CI)	Mean difference (95% CI)
Primary Measures							
Fasting duration (hours)							
Mean Median Range	7.6 ± 8.5 5 1–75	5.8 ± 4.8 4 0–35	6.2 ± 5.6 4 0–28	5.7 ± 4.8 4 0–33	−1.89 (−3.06–0.72)	−1.46 (−2.59–0.33)	−1.94 (−3.04–0.86)
Fasting duration by adherence outcome							
	*n (%)*	*n (%)*	*n (%)*	*n (%)*	*Relative risk ratio* *(95% CI)*	*Relative risk ratio* *(95% CI)*	*Relative risk ratio* *(95% CI)*
Good (1‐2 h)	22.0 (10.0)	32.0 (16.3)	30.0 (13.5)	44.0 (16.7)	1.76 (0.98, 3.14)	1.41 (0.78, 2.52)	1.81 (1.05, 3.12)
Average (3‐5 h)	89.0 (40.5)	85.0 (43.4)	98.0 (44.1)	110.0 (41.8)	1.13(0.76, 1.67)	1.16 (0.80, 1.70)	1.06 (0.74, 1.52)
Poor (> 6 h)	109.0 (49.5)	79.0 (40.3)	94.0 (42.3)	109.0 (41.4)	0.69 (0.47, 1.02)	0.75 (0.51, 1.09)	0.72 (0.50, 1.03)
Secondary measures IV fluid therapy administered							
	*n (%)*	*n (%)*	*n (%)*	*n (%)*	*Odds ratio* *(95% CI)*	*Odds ratio* *(95% CI)*	*Odds ratio* *(95% CI)*
Preoperatively	94.0 (42.7)	84.0 (42.9)	97.0 (43.7)	107.0 (40.7)	1.01 (0.68, 1.48)	1.04 (0.71, 1.52)	0.92 (0.64, 1.32)
Intraoperatively	111.0 (50.5)	104.0 (53.1)	105.0 (47.3)	105.0 (39.9)	1.11 (0.76, 1.63)	0.88 (0.61, 1.28)	0.65 (0.46, 0.94)
Postoperatively	24.0 (10.9)	16.0 (8.2)	17.0 (7.7)	28.0 (10.6)	0.73 (0.37, 1.41)	0.68 (0.35, 1.30)	0.97 (0.55, 1.73)
IV fluid bolus administered							
	*n (%)*	*n (%)*	*n (%)*	*n (%)*	*Odds ratio* *(95% CI)*	*Odds ratio* *(95% CI)*	*Odds ratio* *(95% CI)*
Preoperatively	12.0 (5.5)	5.0 (2.6)	4.0 (1.8)	12.0 (4.6)	0.45 (0.16 1.31)	0.32 (0.10, 1.00)	0.83 (0.37, 1.88)
Postoperatively	3.0 (1.4)	0 (0)	2.0 (0.9)	1.0 (0.4)	1.000	0.66 (0.11, 3.97)	0.28 (0.03, 2.67)

*Note:* Plus–minus values mean Standard Deviation (±SD). ***N.B***. Linear mixed‐effects model for fasting duration includes patient identifier as random intercept. Binary logistic and multinomial logistic regressions assess odds of fluid/bolus administration and fasting category across timepoints. All models include timepoint as a fixed effect only.

**FIGURE 3:**
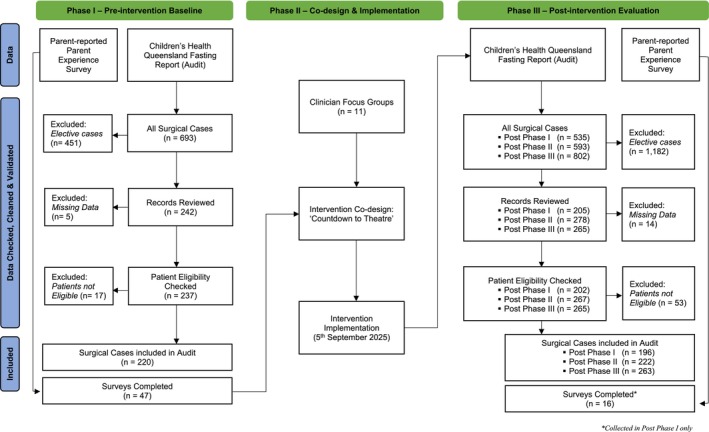
Flow of participant inclusion and data collection across study phases.

The intervention did not result in changes to the administration of preoperative and postoperative IV therapy or fluid boluses. There was, however, a reduction in intraoperative IV therapy administered at 6 months postintervention implementation (Post‐Phase III).

### 3.4 Patient Experience of Preoperative Fasting

The results of the parent‐reported patient experience survey (summarised in Table 4) indicate mixed feedback, but an overall improvement in many areas. An increase in 5‐star satisfaction ratings postimplementation was evident (44.7%–68.8%). Similarly, despite a slight increase in reports of excessive crying or irritability postimplementation (23.3%–27.3%), parents in the postimplementation phase reported that their children were less upset about not being allowed to eat or drink (23.3%–15.22%), and were more calm, content or happy (12.6%–18.2%). There was also an improved attitude towards the duration of the fasting experience, with more reports stating that the duration of fast was ‘the perfect length of time’ (6.3%–18.75%), and less perceptions of excessive (29.2% to 25%) or uncomfortable but manageable (56.3% to 50.0%) fasting experiences.

**TABLE 4:** Parent‐reported patient experience survey responses.

	Preintervention *n* (%)	Postintervention *n* (%)
Overall, how satisfied were you with your child's fasting experience?		
★	2 (4.3)	
★★	1 (2.1)	
★★★	6 (12.8)	3 (18.8)
★★★★	17 (36.1)	2 (12.5)
★★★★★	21 (44.7)	11 (68.8)
How did you feel about how long your child had to fast for surgery?		
It was too long	14 (29.2)	4 (25.0)
It was uncomfortable but manageable	27 (56.3)	8 (50.0)
It was the perfect length of time	3 (6.3)	3 (18.75)
It was shorter than expected	3 (6.3)	
No response	0 (0.0)	1 (6.25)
During the fasting period, did you child display any of the following behaviours? *(Multiselect)*		
Excessive crying/irritability	24 (23.3)	9 (27.3)
Repeatedly asking for food/drink	26 (25.2)	8 (24.2)
Being upset about not being allowed to eat/drink	24 (23.3)	5 (15.2)
Poor compliance to instructions	4 (3.9)	2 (6.1)
Easily distractible (e.g., toys, technology)	12 (11.7)	3 (9.1)
Calm/content/happy	13 (12.6)	6 (18.2)
Were you given clear instructions on fasting requirements?		
Yes	44 (93.6)	15 (93.8)
No	3 (6.4)	1 (6.2)
Were you informed of the risks associated with not following fasting instructions?		
Yes	29 (61.7)	11 (68.7)
No	18 (38.3)	5 (31.3)
Were you informed of the possibility of a prolonged theatre wait time?		
Yes	27 (57.4)	6 (37.5)
No	7 (14.9)	1 (6.2)
No response	13 (27.7)	9 (56.3)
How satisfied were you with the information you were told or given regarding theatre wait times?		
★		
★★	3 (6.4)	
★★★	12 (25.5)	4 (25.0)
★★★★	10 (21.3)	2 (12.5)
★★★★★	22 (46.8)	10 (62.5)
How often were you updated by nursing staff about your child's procedure start time?		
Less than hourly	6 (12.8)	5 (31.2)
Hourly	16 (34.0)	3 (18.8)
Every 2–3 h	13 (27.7)	2 (12.5)
Every 4–5 h	2 (4.3)	
Greater than every 5 h	1 (2.1)	
Never	4 (8.5)	3 (18.8)
Not sure/other	5 (10.6)	3 (18.8)
How satisfied were you with how often you were updated about your child's procedure start time?		
★	1 (2.1)	
★★	3 (6.4)	1 (6.2)
★★★	9 (19.1)	3 (18.8)
★★★★	14 (29.8)	1 (6.2)
★★★★★	20 (42.6)	11 (68.8)

There were mixed results regarding the quality of the information provided, impacting patient fasting experience. No change was observed in the clarity of information provided regarding fasting requirements, with consistently high rates of 93.6% preimplementation and 93.8% postimplementation. Although most parents still felt they were informed of the possibility of a prolonged wait time, this metric decreased from 57.4% to 37.5% postimplementation, suggesting a need for further emphasis on communicating the potential of delays to patients and families. There was an improvement in the proportion of parents who reported being informed of the risks associated with not following fasting instructions, increasing from 61.7% to 68.7%. Overall satisfaction with the information provided, as demonstrated by the incidence of the highest satisfaction rating of 5 stars, increased from 46.8% to 62.5% postimplementation. This demonstrates that despite the mixed results, the overall perception of information quality improved postimplementation.

## 4 Discussion

While previous studies have sought to address prolonged preoperative fasting through a variety of interventions (Dulay et al. 2024), its persistent prevalence in practice underscores a critical need to leverage implementation science frameworks to more effectively bridge the evidence–practice gap (Bauer and Kirchner 2020). These frameworks ensure that patients can fully benefit from advancements in healthcare research, by enhancing an intervention's adoption, impact, effectiveness and sustainability in clinical practice (Grimshaw et al. 2012). Consequently, this study utilised the COM‐B framework to explore the behavioural drivers underpinning current fasting practices (Michie et al. 2011). Among several barriers—such as poor‐real time monitoring of fasting duration, fear of noncompliance and a systemic pressure to avoid theatre delays—the primary driver perpetuating prolonged fasting was the inconsistent communication between bedside and theatre clinicians, which compromised the timely offering of clear fluids. Importantly, a liberal clear fluid fasting protocol was already in place, supported by a culture of patient‐centred care and strong leadership committed to quality improvement. These facilitators provided a foundation for the co‐design and implementation of the ‘Countdown to Theatre’ intervention.

Implementation of this intervention demonstrated an overall trend of improvement in both fasting adherence and patient experience. This was evidenced by a leftward shift in the distribution of fasting duration (Figure 2), reflecting a greater proportion of patients fasting for optimal durations in the postimplementation phases. Although statistical significance was not measured, the sustained improvement over a 6‐month period suggests meaningful clinical significance—an outcome that distinguishes this intervention from many previous efforts, many of which neither evaluated nor successfully demonstrated sustainability (Dulay et al. 2024). Importantly, no changes were observed in the use of preoperative or postoperative intravenous therapy across study phases, indicating that improvements were not attributable to alterations in clinical management. Patient and family experience also improved, with higher satisfaction reported in relation to the quality of information provided, emotional well‐being of fasting children and overall fasting experience.

The success of the quality improvement project hinged on several key factors. Collaboration through co‐design with key stakeholders proved instrumental, as their insights into the barriers and facilitators contributing to prolonged fasting ensured that the intervention design effectively targeted the most critical issues. Active stakeholder engagement not only fostered a collaborative willingness to develop a cohesive and unified approach but also facilitated clear communication within their respective discipline groups, ensuring alignment and consistency across teams. Effective communication and education tools, including verbal methods such as in‐services, written materials, and visual prompts like bedside posters and communication boards, alongside the accessibility of project leaders, further supported the dissemination and reinforcement of essential information. Moreover, a strong emphasis on actively listening to and incorporating feedback during early implementation proved to be a key determinant of success, allowing swift and effective adjustments to address challenges as they emerged.

Building upon the collaborative efforts and feedback‐driven approach, the intervention itself showcased a well‐structured and adaptable design that effectively addressed the challenges of prolonged fasting while balancing operational efficiency, patient safety and experience. By empowering bedside nurses with clear responsibility for managing patient fasting, the intervention ensured that patients either had access to clear fluids at least every 2 h or stopped drinking only when imminently scheduled for theatre. This shift not only enhanced the consistency of fasting adherence but also positioned the nursing workforce as central to driving evidence‐based practice change. This nurse‐led model highlights the critical role of the nursing profession in operationalising research, improving care delivery and leading sustainable, system‐level improvements that directly benefit patients and the broader healthcare system.

Recognising the complexity of the healthcare system and the challenges of applying a one‐size‐fits‐all approach, this intervention provided a structured yet flexible solution. Importantly, unlike numerous previous studies that focused solely on patients scheduled for elective procedures (Dulay et al. 2024), this intervention demonstrated its applicability and effectiveness in addressing the unique challenges associated with emergency cases. While adaptable to evolving guidelines, the intervention alone cannot address broader cultural and systemic barriers to fasting adherence, such as waiting times for theatre, which continue to impact the perioperative process. Further efforts are needed to tackle these larger systemic issues to fully optimise patient outcomes.

## 5 Limitations

Despite the progress achieved through the quality improvement project, certain challenges persist, with approximately 40% of patients still experiencing fasting durations exceeding 6 h. Further investigation is warranted to identify the contributing factors, which may include overnight fasting, clinical interventions by medical teams overriding nursing protocols, or individual patient characteristics, such as children refusing to drink clear fluids despite being offered. While the interventions demonstrated clinical significance through positive trends in reducing excessive fasting durations and narrowing variability, none of the risk or odds ratios achieved statistical significance. Future studies should address this limitation by incorporating larger, powered sample sizes to ensure more robust statistical analyses (Althubaiti 2023).

Although the patient surveys revealed interesting trends, the postintervention sample size was too small to draw definitive conclusions or claim substantial improvements. Statistical analyses comparing the pre‐ and postcohorts were not performed, reflecting the quality improvement nature of the project, which was not hypothesis‐driven. Additionally, survey data collection did not occur at 6 months post intervention, a point at which effects may have been more sustained. This underscores the necessity of larger cohorts, comprehensive statistical evaluation, and extended follow‐up periods in future research.

Another limitation of this study is the possible lack of generalisability outside of local workflows and culture, as the intervention was specifically tailored to address the unique barriers and facilitators identified within this setting. The co‐designed approach, while effective in optimising processes and outcomes at QCH, may not directly translate to other institutions with different operational workflows, organisational cultures, or patient populations. To ensure broader applicability, future studies should consider adapting and evaluating the intervention in diverse healthcare settings, accounting for local barriers and facilitators to preoperative fasting adherence.

## 6 Conclusion

The ‘Countdown to Theatre’ intervention demonstrates the feasibility and effectiveness of co‐designed strategies in improving adherence to preoperative fasting guidelines while enhancing the overall patient and family experience. While this study focused on addressing specific challenges within QCH, its principles of co‐design, structured implementation and application of the COM‐B framework provide a replicable model for addressing similar challenges in other healthcare settings. Future research should explore opportunities to expand this intervention to diverse healthcare environments and further refine its components to address systemic barriers. By doing so, this approach can contribute to advancing patient safety, experience and adherence to fasting protocols across paediatric healthcare.

## Ethics Statement

The project was reviewed by the Queensland Children's Hospital Human Research Ethics Committee prior to commencement and was granted an exemption under the terms of quality improvement (EX/22/QCHQ/84401). There is a statistician on the author team by name Diana Hermith‐Ramirez.

## Conflicts of Interest

The authors declare no conflicts of interest.

## Supporting information


Data S1.

## Data Availability

The data that support the findings of this study are available on request from the corresponding author. The data are not publicly available due to privacy or ethical restrictions.
