# Supplementary material for: Implementation of the ‘Countdown to Theatre’ Approach to Bridge the Evidence–Practice Gap in Paediatric Preoperative Fasting: A Quality Improvement Initiative
Source: J Adv Nurs. 2025 Aug 27;82(5):5405–18. doi: 10.1111/jan.70162 (PMC13069189; doi:10.1111/jan.70162)

SUPPLEMENTARY FIGURE A – Child and Family Education Resource

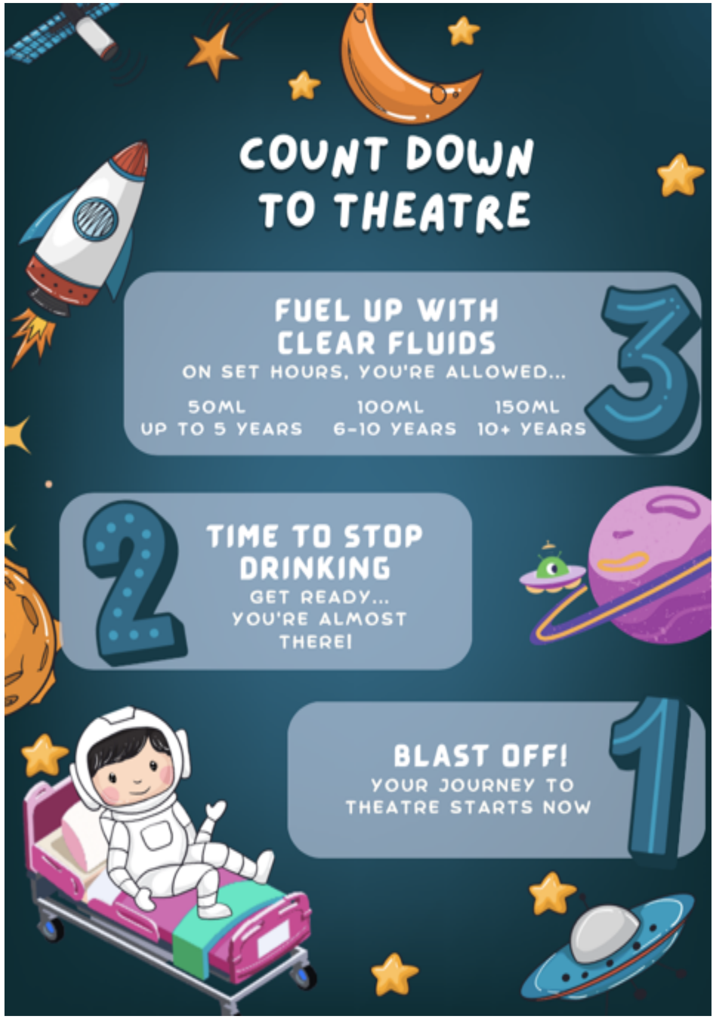

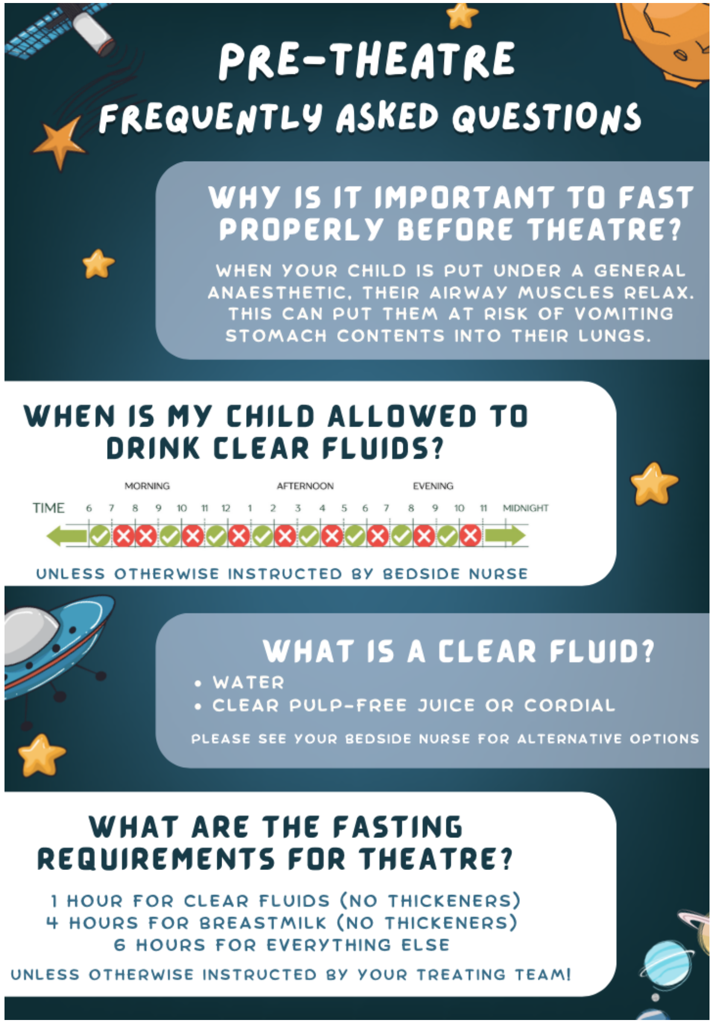

SUPPLEMENTARY FIGURE B – Clinical Area Communication Board

Preoperative Fasting Board

Emergency Board Patients ONLY

QCH Surgical Division

**Patient Name**	**Bed No.**	**Amount Restriction**	**Fasting Stage**	0600-0659	0900-0959	1100-1159	1300-1359	1500-1559	1700-1759	1900-1959	2100-2159	2300-0600

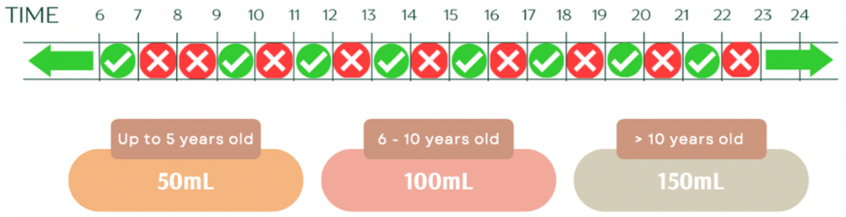

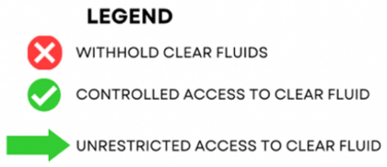

Supplement: Supplementary file 1 — Data S1. [file JAN-82-5405-s001.pdf]
